# Allen & Masser

**Published:** 1882-01-20

**Authors:** 


ALLEN & MASSER—PHARMACEUTICAL CHEMISTS.—We ask special attention to the advertisement of this excellent house in the present issue of our Journal.

